# Association of DNA methylation age acceleration with digital clock drawing test performance: the Framingham Heart Study

**DOI:** 10.18632/aging.206285

**Published:** 2025-07-21

**Authors:** Zexu Li, Huitong Ding, Mengyao Wang, Yi Li, Ting Fang Alvin Ang, Gurnani Ashita, Katherine A. Gifford, Cody Karjadi, Daniel Levy, Rhoda Au, Chunyu Liu

**Affiliations:** 1 Department of Anatomy and Neurobiology, Boston University Chobanian and Avedisian School of Medicine, Boston, MA 02118, USA; 2 Framingham Heart Study, Boston University Chobanian and Avedisian School of Medicine, Boston, MA 02118, USA; 3 Department of Biostatistics, Boston University School of Public Health, Boston, MA 02118, USA; 4 Slone Epidemiology Center, Boston University Chobanian and Avedisian School of Medicine, Boston, MA 02118, USA; 5 Population Sciences Branch, Division of Intramural Research, National Heart, Lung, and Blood Institute, National Institutes of Health, Bethesda, MD 20892, USA; 6 Department of Neurology, Boston University Chobanian and Avedisian School of Medicine, Boston, MA 02118, USA; 7 Department of Epidemiology, Boston University School of Public Health, Boston, MA 02118, USA; 8 Department of Medicine, Boston University Chobanian and Avedisian School of Medicine, Boston, MA 02118, USA

**Keywords:** epigenetic aging, DNA methylation, cognitive function, digital Clock Drawing Test

## Abstract

Background: The relationship between cognitive function, measured by digital Clock Drawing Test (dCDT), and biological aging is lacking.

Methods: We used linear mixed regression to evaluate the associations between epigenetic aging metrics (Horvath, Hannum, GrimAge, PhenoAge, DunedinPACE) and dCDT scores in the Framingham Heart Study (FHS), adjusting for covariates. Significance was set at a false discovery rate (FDR) <0.05.

Results: Among the 1,789 FHS participants (mean age 65 ± 13, 53% women), higher epigenetic age acceleration metrics at baseline predicted lower dCDT scores approximately seven years later. The magnitude of these associations was greater in older participants (≥65 years, *n* = 985). The strongest association was observed between the dCDT total score and DunedinPACE in the full sample (beta = −2.1, FDR = 0.0004), the younger (<65 years; beta = −1.9, FDR = 0.02), and older (beta = −2.2, FDR = 0.01) age groups. Additionally, the dCDT total score was associated with age acceleration estimated by Horvath (beta = −1.9, FDR = 0.01) and PhenoAge (beta = −2.5, FDR = 0.01) in older participants, while not in the full sample or younger participants. Furthermore, higher levels of DNAm-based PAI1 (beta = −0.9, FDR = 0.005) and ADM (beta = −2.9, FDR = 0.01), components of GrimAge, were significantly associated with lower dCDT total scores. In analyses of cognitive subdomains, simple motor function was significantly associated with DunedinPACE (FDR = 0.005) in both age groups, and with GrimAge (FDR = 0.05) in the older age group, suggesting that deterioration in various organ systems may particularly impact this domain.

Conclusion: Our findings suggest that advanced biological aging, particularly as captured by DunedinPACE and GrimAge components, is significantly associated with poorer cognitive performance measured by dCDT, especially in older adults, highlighting a potential link between systemic aging processes and cognitive decline.

## INTRODUCTION

Cognitive function, representing a crucial aspect of overall brain health, encompasses various domains such as memory, reasoning, and attention [1]. Neuropsychological (NP) tests are typically used to measure cognitive functions for individuals, focusing on one or several specific cognitive domains. For example, the long−used Clock Drawing Test (CDT) evaluates executive functioning and spatial skills [2], and has been typically conducted with pen on paper and assessed with one overall score and visual inspection by the clinician. However, the interpretation of NP tests often relies on clinical expertise such as a neuropsychologist or neurologist, who are not readily available in non−urban centers in the U.S. or many countries globally. Further clinical interpretation is open to subjective bias and is not uniformly consistent. The use of digital devices in NP tests is gaining recognition for their efficiency, precision, automation, and reproducibility [3]. The digital Clock Drawing Test (dCDT), a digital version of the standardized CDT done with pen and paper, provides a much more robust assessment of cognitive functioning, with an overall score but also quantitative markers of sub−domains including motor function, drawing efficiency, spatial reasoning, and information processing [4]. Previous research shows comparable performance between dCDT and other NP tests, which can identify mild cognitive impairment [5], and is associated with markers of neurodegeneration, such as brain volumes [6]. Compared to CDT, dCDT appears to capture more subtle cognitive changes [7].

Aging is an inevitable process for humans, resulting in a decline in physiological capacity and an increasing risk of various disease conditions [8], including neurodegenerative diseases [9]. Biological aging, the changes at the molecular level [10, 11], is essential to understand the heterogeneity in healthy aging [12]. Epigenetic modifications, such as DNA methylation (DNAm), measure biological aging at the epigenetic level [13–15]. DNAm age [16] has been associated with general health [17, 18] and neurodegenerative diseases [19]. These aging metrics have also shown predictive value for the development of diabetes [20], mortality [21], cognitive dysfunction [22, 23], and physical aging [23]. The first generation of epigenetic clocks (i.e., Horvath and Hannum clocks) was developed to estimate chronological age using DNAm levels at specific 5′−C−phosphate−G−3′ (CpG) sites [16, 24]. The second generation of epigenetic clocks (e.g., GrimAge and PhenoAge) was designed to predict mortality using CpG sites associated with clinical markers, such as plasma protein levels [25–27]. Subsequently, third−generation epigenetic clocks, like DunedinPACE, measure how fast a person is biologically aging rather than estimating their age. They use long−term health data to better predict future health and lifespan [28]. Among them, GrimAge estimates biological age by using DNAm markers to infer levels of aging−related plasma proteins and smoking history [27]. To improve reliability, PC−based versions of first− and second−generation clocks have been developed, reducing technical noise by summarizing methylation signals into principal components [29]. DNAm age acceleration is assessed by regressing the estimated DNAm age on chronological age [30].

Studies have established correlations between traditional cognitive assessments and DNAm age acceleration [22, 23, 31–35], showing the possibility that DNAm age acceleration is a risk factor or marker for cognitive decline. However, research on the relationship between cognitive function measured by digital devices and DNAm age acceleration is currently lacking. Our study aimed to fill this gap. We hypothesize that DNAm aging at baseline predicts cognitive status measured at a later time point. To test our hypothesis, in the primary analysis, we investigated the associations of overall cognitive function, measured by dCDT with five DNAm−based age acceleration metrics in the Framingham Heart Study (FHS). In the secondary analysis, we investigated the associations of sub−domain functions measured by dCDT with five DNAm−based age acceleration metrics. We further investigated the associations between dCDT and DNAm−based plasma protein surrogates incorporated into GrimAge. All analyses were conducted on the full sample, the younger (<65 years) and the older (≥ 65 years) age groups. We seek to gain additional insights into the underlying molecular mechanism of cognitive function measured by digital devices and investigate how different aging metrics are related to cognitive functions.

## RESULTS

### Participant characteristics

This study included 1,789 participants in FHS (mean age 65 ± 13 at dCDT, 53% women) (Table 1). On average, DNAm was measured seven years before the dCDT measurement (Supplementary Figure 1). Education levels were significantly higher in the younger age group (<65 years) compared to the older age group (≥65 years). On average, participants in the younger age group exhibited higher dCDT total score and sub−domain scores compared to those in the older age group (all *p* < 0.001). The average estimated DNAm ages differed between DNAm metrics (Supplementary Table 1). For instance, the mean Hannum age was estimated as 62, while the mean Horvath age was estimated as 53 in the full samples. Compared to the mean chronological age, the mean DNAm ages calculated by Hannum, GrimAge, and DunedinPACE showed acceleration (with DNAm age higher than chronological age) in the full sample, whereas PhenoAge and Horvath showed lower mean DNAm ages than the mean chronological age. We further observed that, except for DunedinPACE, males tended to have advanced DNAm ages compared to females (Supplementary Table 2).

**Table 1:** Demographic and clinical characteristics of study participants.

	**Total**	**Age <65**	**Age ≥65**	***P*−value**
	**(*n* = 1789)**	**(*n* = 804)**	**(*n* = 985)**	
**Female, *n* (%)**	955 (53%)	407 (51%)	548 (56%)	0.208
**Age at dCDT (years)**	65 (± 13)	54 (± 8)	75 (± 7)	
**Age at DNAm (years)**	58 (± 12)	48 (± 7)	67 (± 7)	
**Education levels, *n* (%)**				
Incomplete high school	30 (2%)	6 (1%)	24 (2%)	<0.001
High school graduate	332 (19%)	108 (13%)	224 (23%)	
Some college	556 (31%)	231 (29%)	325 (33%)	
College graduate or above	871 (49%)	459 (57%)	412 (42%)	
**Dementia**	51 (3%)	0	51 (5%)	<0.001

In this study, we used age at DNAm for age group stratification. Mean with standard deviation (SD) was provided for a continuous variable, while count and proportion were provided for categorical variables.

### Association between dCDT total score and DNA methylation age acceleration

**Figure 1:**
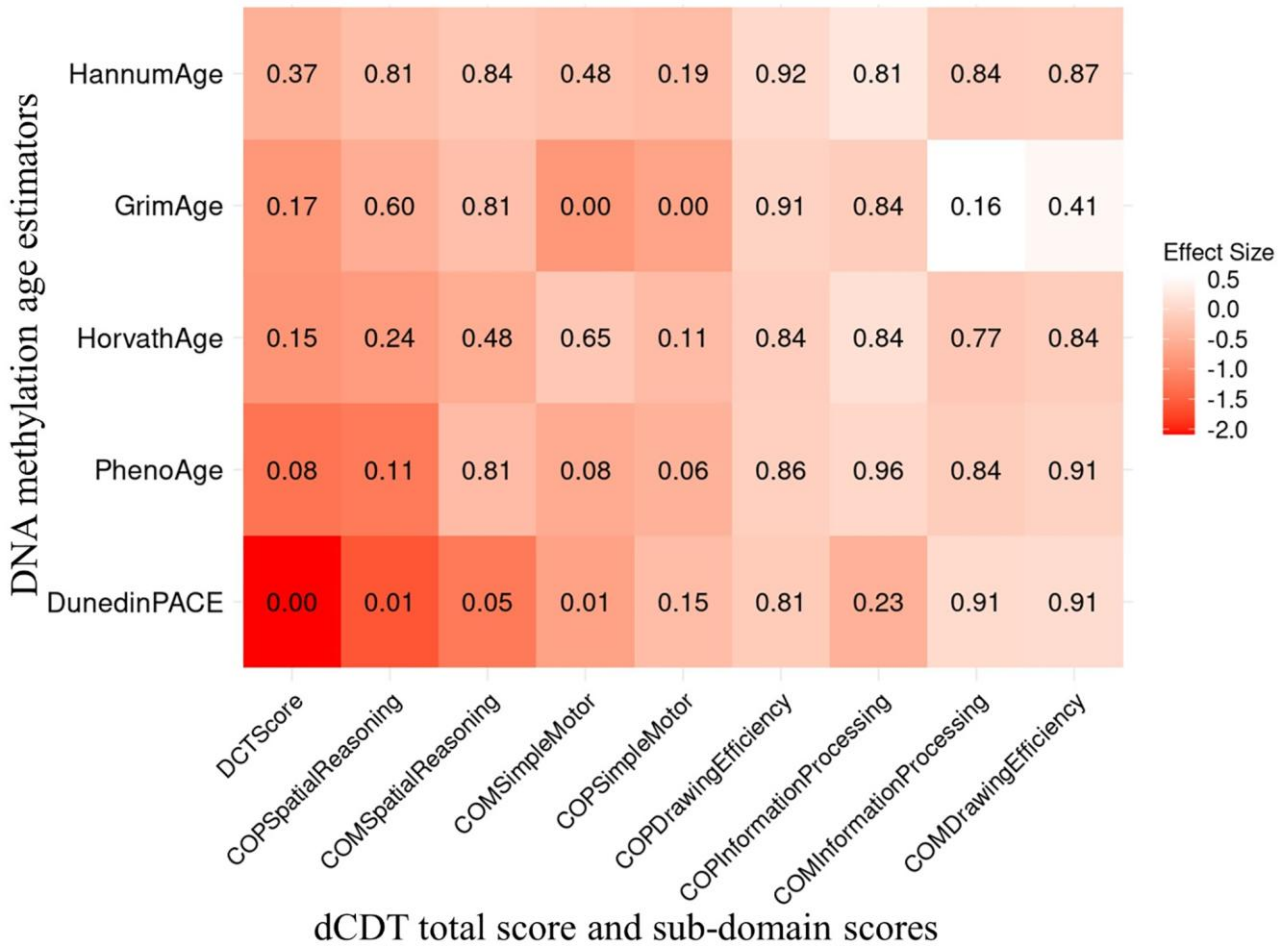
**Association between dCDT scores and DNAm age acceleration in 1789 participants of the FHS.** The dCDT total score includes command task composite scores and copy task composite scores. DNA methylation age acceleration was obtained by regressing DNAm age metrics on chronological age. We conducted association analysis between standardized DNAm age acceleration and the dCDT total score, adjusted for age, self−reported sex, education and cell counts. The numbers inside each cell represent the P−values of the associations. The color represents the change in dCDT scores corresponding to a one SD increase in DNAm age acceleration.

**Figure 2:**
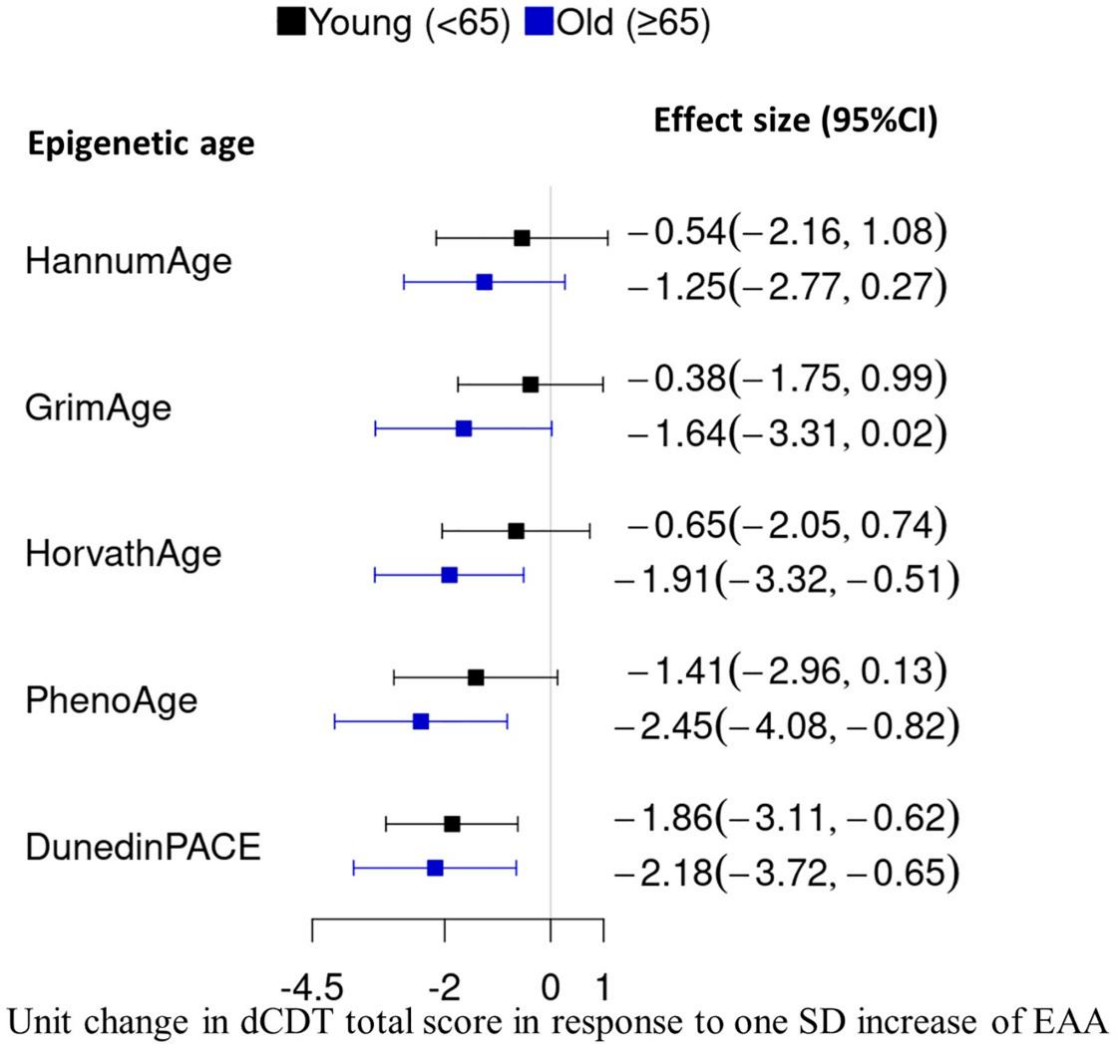
**Comparison of effect size in the association between DNAm age acceleration and the dCDT total score.** DNAm age acceleration was obtained by regressing DNAm metrics on chronological aging, followed by standardization with a mean of zero and SD of one. We conducted an association analysis between the dCDT total score and standardized DNAm age acceleration. CI, confidence interval.

The primary association analysis examined the associations between dCDT total score and several DNAm age acceleration metrics, including PC−based versions of first− and second−generation clocks [29], as well as DunedinPACE [28]. Significance was assessed at False Discovery Rate (FDR) <0.05. In the full sample, a higher dCDT total score was associated with lower DNAm age acceleration estimated by all DNAm aging metrics (Figure 1). For example, a one−SD higher level in the pace of aging (DunedinPACE) was associated with a 2.1−unit lower level in the dCDT total score (FDR = 0.0004) (Figure 1). Although lower total scores were associated with higher DNAm age acceleration across the other metrics, these associations were not statistically significant in the full sample (Figure 1). Age showed a significant effect modification of the association (*p* = 0.004) with DunedinPACE but not for other epigenetic age metrics (Supplementary Table 3). Thus, we conducted stratified analyses by age groups for all DNAm age metrics. In the younger age group, DunedinPACE was the only DNAm metric associated with the dCDT total score (beta = −1.9, FDR = 0.02). In contrast, in the older age group, three DNAm aging metrics were significantly associated with the dCDT total score: DunedinPACE (beta = −2.2, FDR = 0.01), DNAm age acceleration estimated by Horvath (beta = −1.9, FDR = 0.01) and PhenoAge (beta = −2.5, FDR = 0.01). Although the association between dCDT total score and other DNAm aging metrics was not significant, the directionality of the associations was consistent in both older and younger age groups (Figure 2 and Supplementary Figures 2, 3).

We conducted multiple secondary analyses to examine the robustness of our primary results. We first tested the association between DNAm age acceleration estimated by GrimAge version 2 and dCDT total score. Similar effect sizes and association direction were observed with DNAm age acceleration estimated by PC−based GrimAge version 1 (Supplementary Figure 4). Additionally, GrimAge version 2 showed a stronger association with dCDT total score in the older age group (beta = −2.0, FDR = 0.02) compared to PC−based GrimAge version 1 (beta = −1.6, FDR = 0.07) (Supplementary Figures 4–6). We further assessed if associations between dCDT total score and DNAm aging metrics differed by self−reported sex. No significant sex differences were observed in the association between dCDT total score and DNAm aging metrics (Supplementary Table 4). However, given its relevance as a biological variable, we conducted a sex−stratified analysis to explore potential variations. We observed similar effect sizes and directions of association between dCDT scores and all DNAm age acceleration measures in men and women. One minor difference was that DunedinPACE showed a stronger association with dCDT total score in males (beta = −2.3, FDR = 0.01) compared to females (beta = −1.7, FDR = 0.06) (Supplementary Figures 7 and 8).

### Association between dCDT sub−domain scores and DNA methylation age acceleration

In additional secondary analyses, we investigated the association between dCDT sub−domain scores and several DNAm age acceleration metrics, including PC−based versions of first− and second−generation clocks [29], as well as DunedinPACE [28]. In the full sample, we observed a significant association between DunedinPACE and both the dCDT simple motor function score in the command task (FDR = 0.01) and the spatial reasoning score from the copy task (FDR = 0.01). A one−SD higher level in the pace of aging was associated with a 0.7−unit decrease in the dCDT simple motor function score and a 1.6−unit decrease in the spatial reasoning score. DNAm age acceleration estimated by GrimAge was also found to be significantly associated with the dCDT simple motor function score in both the copy task (beta = −0.7, FDR = 0.005) and command task (beta = −0.9, FDR = 0.005) in the full sample. No other significant association was found between the other epigenetic age acceleration metrics and the dCDT sub−domain score in the full sample (Figure 1).

Different from the result in the full sample, we only observed similar results for DNAm age acceleration estimated by GrimAge in the older age group, where a one−SD increase in GrimAge acceleration was significantly associated with a 1.1−unit decrease in dCDT simple motor function score in the command task (FDR = 0.04) (Supplementary Figure 2). In addition, DNAm age acceleration estimated by PhenoAge was significantly associated with the dCDT simple motor function score in the command task (beta = −1.0, FDR = 0.04). While no significant association was found between dCDT sub−domain scores and DNAm aging metrics in the younger age group, the directionality was consistent for most associations in both younger and older age groups (Supplementary Figures 2, 3).

### Association between dCDT scores and plasma protein levels

To gain insight into biological mechanisms underlying the cognitive functions, we examined the association between dCDT scores and the seven DNAm−based plasma protein levels incorporated into GrimAge version 1 [25], including Cystatin C, B2M, GDF15, TIMP1, Leptin, PAI1, and ADM. In the full sample, dCDT total score was significantly associated with DNAm−based PAI1 (beta = −0.9, FDR = 0.005) and ADM (beta = −2.9, FDR = 0.01). Although no significant associations were observed between dCDT total score and other DNAm−based plasma protein levels, all associations showed a negative direction, indicating that higher DNAm−based protein levels corresponded to lower dCDT total score (Supplementary Figure 9).

Among sub−domain scores, several DNAm−based proteins showed significant associations (Supplementary Figure 9). Cystatin C was associated with simple motor function in the command task (beta = −0.7, FDR = 0.03). GDF15 was associated with simple motor function in both the command (beta = −0.8, FDR = 0.01) and copy task (beta = −0.6, FDR = 0.01). Leptin was associated with spatial reasoning in the copy task (beta = −5.8, FDR = 0.01). PAI1 was significantly associated with spatial reasoning in the copy task (beta = −1.7, FDR = 0.02), simple motor function in the command task (beta = −0.8, FDR = 0.01) and copy task (beta = −0.7, FDR = 0.01). ADM was also associated with spatial reasoning in the copy task (beta = −3.0, FDR = 0.01), simple motor function in the command task (beta = −1.0, FDR = 0.05) and copy task (beta = −1.0, FDR = 0.01) (Supplementary Table 5). In the age−stratified sample, no significant associations were observed in the younger group. In the older age group, dCDT total score remained significantly associated with PAI1 (beta = −2.1, FDR = 0.03), TIMP1 (beta = −2.8, FDR = 0.05), and ADM (beta = −2.8, FDR = 0.05) (Supplementary Figures 10 and 11).

## DISCUSSION

In this study, we investigated the association between cognitive function, measured by the dCDT, and DNAm aging metrics in 1,789 middle−aged and older participants in the FHS.

We found that lower dCDT total scores were consistently associated with advanced biological age quantified by DNAm aging metrics. Among these, DunedinPACE showed significant association with the dCDT total score across the full sample, younger (<65 years), and older (≥65 years) age groups. In contrast, several other DNAm aging metrics showed significant associations with the dCDT total score only in the older participant group. To explore potential mechanisms underlying the relationship between cognitive decline and biological aging, we investigated the associations between dCDT scores and DNAm−based estimators of plasma proteins captured by GrimAge version 1. We found that the dCDT total score was consistently associated with PAI1 and ADM in the full sample and the older age group.

As the need for early diagnosis in the preclinical stage of AD grows, advanced screening tools for cognition are desired to capture subtle cognitive changes [36]. The dCDT has drawn attention for its efficiency, precision, automation, and reproducibility [3]. It has been demonstrated to be effective in distinguishing cognitive impairment from normal function [4, 7]. Additionally, it is comparable to established NP tests, such as the Wechsler Memory Scale and Boston Naming Test, in discriminating between participants with mild cognitive impairment (MCI) [6] and those with normal function. Unlike traditional paper and pen tests, the dCDT captures more granular data, including visuospatial, time−base, and kinematic details, offering a more detailed assessment. Its automated scoring system provides clinicians with objective and interpretable results [4].

DNAm is a crucial epigenetic modification that influences gene expression without altering the underlying DNA sequence [37]. By incorporating different sets of CpG sites, the three generations of epigenetic age metrics offer varied perspectives on biological aging. First−generation clocks were designed to predict chronological age based on CpG sites [16, 24], while the second−generation clocks aimed to predict time to death using clinical markers such as plasma protein levels [25–27]. These models primarily estimate DNAm aging based on a combination of chronological age, clinical markers, and mortality risk [16, 24–27]. The third−generation clock, DunedinPACE, focuses on a set of different clinical markers (representing the progressive decline across multiple organ systems captured by multiple CpG sites) [28]. In our study, DunedinPACE was negatively associated with overall cognitive function in both younger and older age groups, while other age clocks only showed significant results in the older age group. These findings indicate that the dCDT test, which is an indicator of brain health, may reflect the aging rates of multiple organ systems.

The analysis of dCDT sub−domain scores with DNAm age provides additional insights into how epigenetic aging may influence specific cognitive domains. For instance, the simple motor function and spatial reasoning sub−domain scores showed significant associations with DunedinPACE, indicating that these domains may be particularly impacted by the deterioration in various organ systems that are relevant across the lifespan. GrimAge is based on seven plasma protein levels related to various diseases and conditions, including cardiovascular disease (Plasma B2M) and cognitive functions (Plasma B2M, ADM, Cystatin C, and leptin) [25]. In this study, simple motor function was associated with advanced GrimAge acceleration. This indicates that the decline of simple motor functions might be related to abnormal levels of these plasma protein markers and is particularly relevant with older chronological age. Future studies are necessary to investigate the associations of these plasma proteins with cognitive decline. In addition to DNAm age acceleration, examining DNAm−based plasma protein levels in relation to dCDT scores may help elucidate potential biological links between specific proteins and cognitive functions. In both the full sample and the older age group, we observed consistent associations between dCDT total score and PAI1 and ADM. Our findings are consistent with several previous studies. PAI1 has been used as a potential diagnostic marker for AD and cognitive function decline [38], showing that PAI1 was significantly different between the healthy control group, MCI group, and AD group within participants with a mean age of seventy−five [38]. A more recent study suggests that increased PAI1 contributes to brain cell senescence in late−onset AD patients, and overexpression of PAI1 secreted from senescent astrocytes can further induce neuroapoptosis [39]. The effect of PAI1 inhibitor in age−related muscle fiber atrophy may also explain its association with motor function in the full sample [40, 41]. These studies suggest a potential biological link between PAI1 and cognitive function, though the underlying mechanisms remain to be fully elucidated. ADM, on the other hand, has also been associated with cognitive function and age−related memory loss [42]. A previous study showed that normal aging was accompanied by an increase of ADM protein expression [43]. A comparison of ADM levels between individuals with AD and cognitively intact participants showed higher ADM expression in the brains of those with AD compared to age−matched healthy controls [43]. This finding aligns with our results, indicating that elevated plasma ADM protein levels are associated with lower dCDT performance. A previous study showed that midregional pro−adrenomedullin peptide (MR−proADM) may have the predictive power of conversion from MCI to AD [44]. The association between ADM and simple motor function may be potentially explained by its association with vascular function [45]. Further study is needed to fully understand the biological pathways between these proteins and cognitive functions.

Digital cognitive measures displayed stronger associations with most DNAm aging metrics among older compared to younger participants, likely to reflect the cumulative and nonlinear age influences on both brain health and DNAm. This is consistent with our earlier findings of stronger associations between alcohol consumption and epigenetic age metrics [46]. For instance, overall cognitive function exhibited significant associations with PhenoAge in the older age group while not in the younger age group. In contrast, the global dCDT score was associated with DunedinPACE in both age groups, indicating that DunedinPACE might be more sensitive to capturing the subtle influence of variations in the pace of aging on cognitive changes across the lifespan.

Several similar studies have investigated the association of cognitive function measured by traditional methods with DNAm aging metrics [22, 31–34]. Marioni et al. reported that general cognitive ability was associated with Horvath age in participants over the age of seventy [32]. Our findings, using the dCDT, are consistent with this, showing similar associations with total score in older participants. Another study assessed how various cognitive tests (e.g., MMSE, ADAS−Cog−13, MoCA) were associated with DNAm age acceleration in participants with a mean age of seventy−five [31]. They found that the faster pace of aging measured by DunedinPACE correlated with more severe cognitive decline. Our results align with this, showing that worse cognitive function was associated with DunedinPACE, with a larger magnitude in older participants. Furthermore, our findings that higher PhenoAge acceleration and DunedinPACE were associated with poorer performance scores in older participants were also consistent with the previous findings, where they found that higher PhenoAge acceleration and DunedinPACE were associated with worse cognitive performance [31]. One additional study investigated the relationship between biological aging and cognitive function. It found that PhenoAge, GrimAge, and DunedinPACE were significant predictors of cross−sectional cognitive dysfunction in 3581 participants with a mean age of 68. Their study setting was similar to our study, which had a time gap between DNAm sample collection and cognitive testing [22]. Our findings in the PhenoAge, GrimAge, and DunedinPACE align with their results, where higher estimated biological aging was associated with poorer performance in the future dCDT.

Our study has limitation, including a lack of diversity (all participants were non−Hispanic Whites) in our study sample. Further research with diverse groups is needed. Our study has several strengths. We employed the dCDT to measure cognitive function, which is a novel measure for assessing cognitive function. In addition, we employed PC−based clocks, which use principal components to reduce noise and enhance accuracy [29]. To mitigate multiple testing, we applied FDR adjustment, which is more appropriate than Bonferroni correction for the presence of correlated outcome and predictor variables. Our study design included an approximate seven−year gap between DNAm measurement at baseline and dCDT assessment at the later time point. This design provided an opportunity to explore the potential predictive value of DNAm−based biological aging measures for later cognitive function. Several prior studies have demonstrated that baseline DNAm−based biological aging can predict future risks of mortality [21], cognitive dysfunction [22, 23] and physical aging [23], suggesting that earlier measures of biological aging might serve as a predictor of age−related morbidity and mortality later in life. Although one longitudinal study has reported that accelerated biological aging remains relatively stable over time among individuals with cognitive decline [47], other research has highlighted that DNAm patterns may change with aging [48] and in response to environmental factors [49]. Further longitudinal studies that incorporate comorbidities, lifestyle, and environmental factors are needed to clarify how temporal changes in DNAm patterns influence biological aging and its relationship with later−life cognitive outcomes.

In conclusion, our study investigated how digital cognitive function assessed by dCDT relates to biological aging, measured by DNAm. These findings highlight the potential role of DNAm in cognitive function. Further research is needed to uncover the underlying biological pathways behind this association, particularly in more diverse populations.

## METHODS

### Study populations

**Figure 3:**
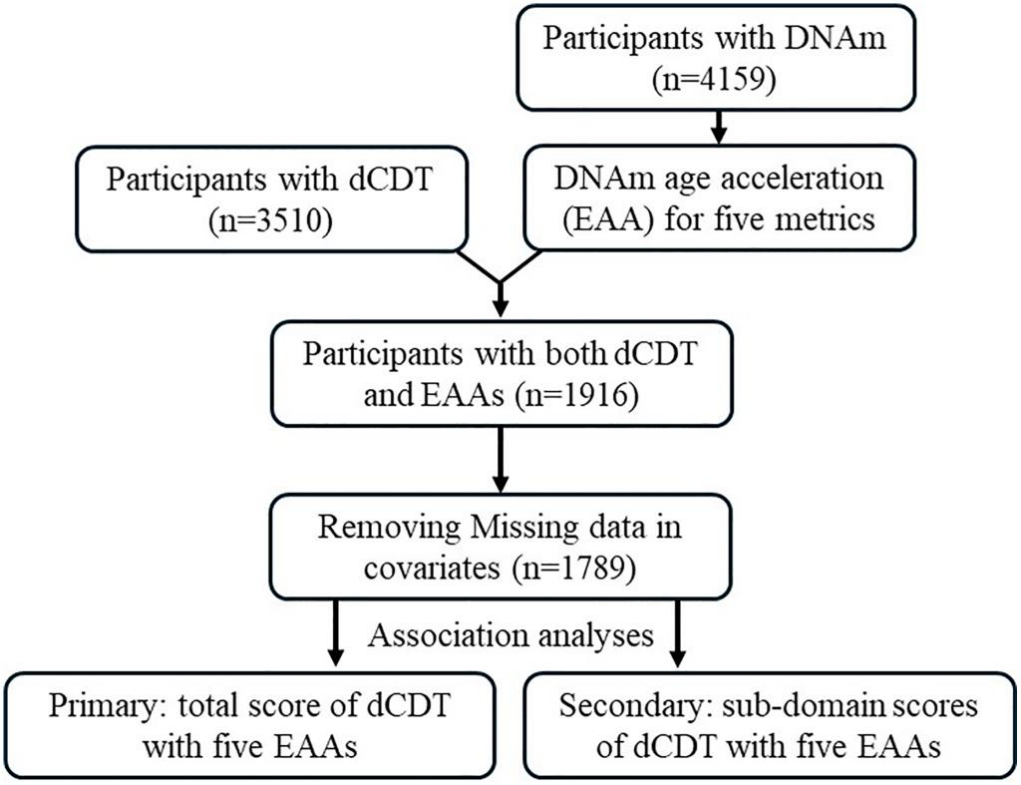
**Flow chart of study design.** The participation of dCDT was solely based on consent. In the FHS, we identified participants with dCDT measurements and DNAm. Five DNAm age metrics were calculated. Epigenetic (DNAm) age acceleration (EAA) was calculated by regression of the DNAm age metrics on chronological age. Primary analysis focused on the association between dCDT total scores and EAAs, whereas secondary analysis focused on the association between dCDT sub−domain scores and EAAs.

FHS, initiated in 1948, is a community−based longitudinal cohort study that has followed three generations of participants over time [50]. All participants underwent routine health assessments every two to six years [51, 52]. Our study included 1,264 participants from the Offspring cohort (Gen2) at exam 8 (2005−2008) and 688 participants from the Third−generation cohort (Gen3) at exam 2 (2008–2011), based on available DNAm measurements. We excluded participants whose blood samples were not collected at exam or who were not administered the dCDT (2011–2018). After excluding participants with covariates (e.g., age, self−reported sex, education, cell counts) missing, we included 1,789 participants (Figure 3) in our statistical analyses. For participants with multiple dCDT tests, we selected dCDT data closest to DNAm measurement dates for inclusion in our study.

### DCTclock

DCTclock serves as an FDA−approved automated screening tool for detecting cognitive changes [4]. Data collection for dCDT using DCTclock involved using a digital pen and specialized paper with a dot matrix grip, which has been previously described in detail [4]. Following the standard protocol, participants were instructed to draw a clock with all the numbers to indicate the time ‘10 after 11’ (command task) and then replicate another clock by copying a provided model (copy task) [53, 54]. Both the drawing process and the final drawing results were recorded, capturing spatial and temporal data, and analyzed through the DCTclock pipeline. These data were treated as input to a trained convolutional neural network to recognize individual symbols with classification probability (e.g., clock face, digits, and small noise stocks). After classifying the individual symbols in drawing, these symbols were used to derive various measurements, such as the correct placement of clock components and pen speed. These measurements were organized into four groups representing different cognitive aspects: Drawing Efficiency, Simple and Complex Motor, Information Processing, and Spatial Reasoning. Drawing efficiency, for instance, evaluated the efficiency in terms of the time spent on drawing and the size of the drawing. Similarly, Simple and Complex Motor measurements represent motor functions, including maximum movement speed. Information Processing focuses on functions like thinking time and latencies, while Spatial Reasoning focuses on spatial abilities through geometric property measurements. A composite score was calculated for each cognitive aspect mentioned above using a Lasso regularized logistic regression model [4], incorporating all the measurements as parameters. Given the performance of two tasks (command and copy), eight sub−domain scores were generated. Additionally, a dCDT total score was computed using a Lasso logistic regression model [4]. Both sub−domain and total scores range from 0 to 100, where a higher score indicates better performance.

### DNAm measurements

DNAm adds a methyl group onto the 5th carbon of cytosine to form 5−methylcytosine [37]. DNAm measurements were conducted using whole blood samples collected during exam 8 for Gen2 and exam 2 for Gen3. DNAm profiling was carried out through a series of procedures, including bisulfite conversion, whole genome amplification, fragmentation, array hybridization, and single−base pair extension [46]. The Illumina Human Methylation 450K Bead chips (Illumina Inc., San Diego, CA, USA) were employed to analyze the DNAm levels across three different laboratories. Detailed information regarding DNAm quantification and quality control procedures in FHS had been previously documented [55].

### DNAm aging metrics

DNAm age is an estimator of aging based on DNAm patterns. Three generations of epigenetic clocks were calculated. The first generation, Horvath’s age [16] and Hannum’s age [24], utilize a set of CpG sites to estimate DNAm age. Horvath’s age calculated a weighted average of 353 clock CpGs with a calibration function to estimate aging from multiple tissues. Hannum’s age considered 71 CpG sites along with a few clinical parameters (self−reported sex, body mass index, etc.) to predict aging in whole blood samples. GrimAge [25], GrimAge version 2 [27] and PhenoAge [26] are the second−generation DNAm aging metrics. Both methods calculated the DNAm age by integrating methylation levels with clinical markers. GrimAge, in the first step, utilized DNAm level, chronological age, and sex to estimate 88 plasma protein levels and smoking pack years with elastic net regression. Then, seven DNAm−based plasma proteins (Cystatin C, B2M, GDF15, TIMP1, Leptin, PAI1, and ADM), age, sex and DNAm−based smoking pack years were selected by elastic net Cox regression model on the prediction of all−cause mortality. Each of the seven DNAm−based plasma protein and smoking pack years was estimated based on fewer than 200 CpG sites. In total, 1,030 unique CpG sites were selected to estimate GrimAge [25, 27]. PhenoAge selected 513 CpGs to predict a linear combination of chronological age and nine clinical markers (e.g., Albumin, White blood cell count), which predicted the time to death [26]. We employed the principal component version of epigenetic clocks (PC−based clocks) to minimize unobserved technical confounders [29]. The DNAm age acceleration for the first− and second−generation aging metrics were residuals calculated by regressing each DNAm age on chronological age. Residuals larger than zero will be considered as accelerated aging. The third generation, DunedinPACE, differed from the previous generations by predicting the pace of aging per year rather than age in years [28]. The pace of aging was calculated from 173 CpGs based on longitudinal change of 19 clinical markers (e.g., blood pressure, total cholesterol, blood urea nitrogen), representing an average rate of biological aging per year of 1−year chronological age [28]. This pace of aging was used as DNAm age acceleration for the following analysis.

### Covariates

Covariates included the age at the dCDT, the time interval between the dCDT and blood sample collection, self−reported sex, educational level, cell count information, and family relationship. The time interval was computed as the age at the dCDT minus the age at blood sample collection. Educational levels were categorized into four groups: less than high school completion, high school graduate, some college, and college graduate. Cell count information was derived from DNAm data. We included the count number of Cytotoxic T cells (CD8+T), B lymphocytes (CD19+ B), granulocytes (Gran), monocytes (Mono), Natural killer cells (NK), and Helper T Cells (CD4+T) as covariates. Family relationships were included as random effects in the model.

### Statistical analysis

The primary analysis explored the relationship between the dCDT total score as the outcome variable and standardized DNAm age accelerations as the predictor variables. The residuals were computed by regressing the DNAm age metrics on chronological age to obtain DNAm age acceleration. The residuals were used as DNAm age acceleration in the following analysis. To facilitate interpretation, we standardized the DNAm age residuals to have a mean of 0 with a standard deviation (SD) of 1. Linear mixed models were employed to assess the association between the dCDT total score and DNAm aging metrics. We adjusted for age at the dCDT, self−reported sex, and educational level and used family as a random effect. We also conducted an age−stratified analysis, dividing participants into two groups based on age at blood sample collection: those younger than 65 years and those aged 65 years or older.

To investigate the association between dCDT sub−domain scores and DNAm aging metrics, linear mixed models were employed with the same set of covariates. The FDR method [56] was applied to adjust for multiple testing [57].

We further investigated the association between dCDT total and sub−domain scores and DNAm−based plasma protein levels estimated using GrimAge version 1. Linear mixed models were employed with the same set of covariates. The FDR method [56] was applied to adjust for multiple testing [57].

## Supplementary Materials

Supplementary Figures

Supplementary Tables
