# Supplementary material for: Association of DNA methylation age acceleration with digital clock drawing test performance: the Framingham Heart Study
Source: Aging (Albany NY). 2025 Jul 21;17(7):1721–45. doi: 10.18632/aging.206285 (PMC12339028; doi:10.18632/aging.206285)
Supplement: Supplementary Figures [file aging-17-206285-s001.pdf]

SUPPLEMENTARY FIGURES

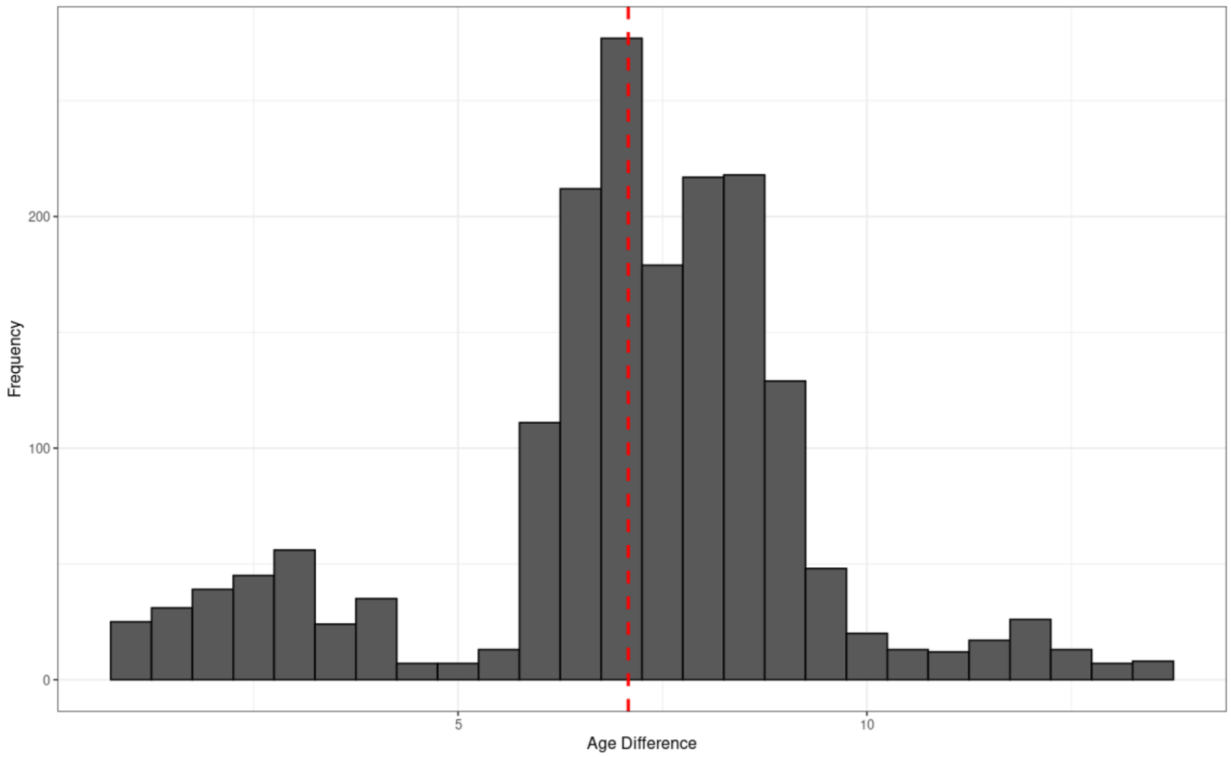

Supplementary Figure 1. Distribution of delta age between DCTclock and DNAm. The x−axis shows the delta age between participants' ages at the digital clock drawing test and when the whole blood sample is collected. Specifically, the delta age is calculated as the difference between the age at DCTclock and the age at DNAm. The y−axis shows the frequency of different delta age values. The red dashed line represents the mean delta age, which is 7.1 years.

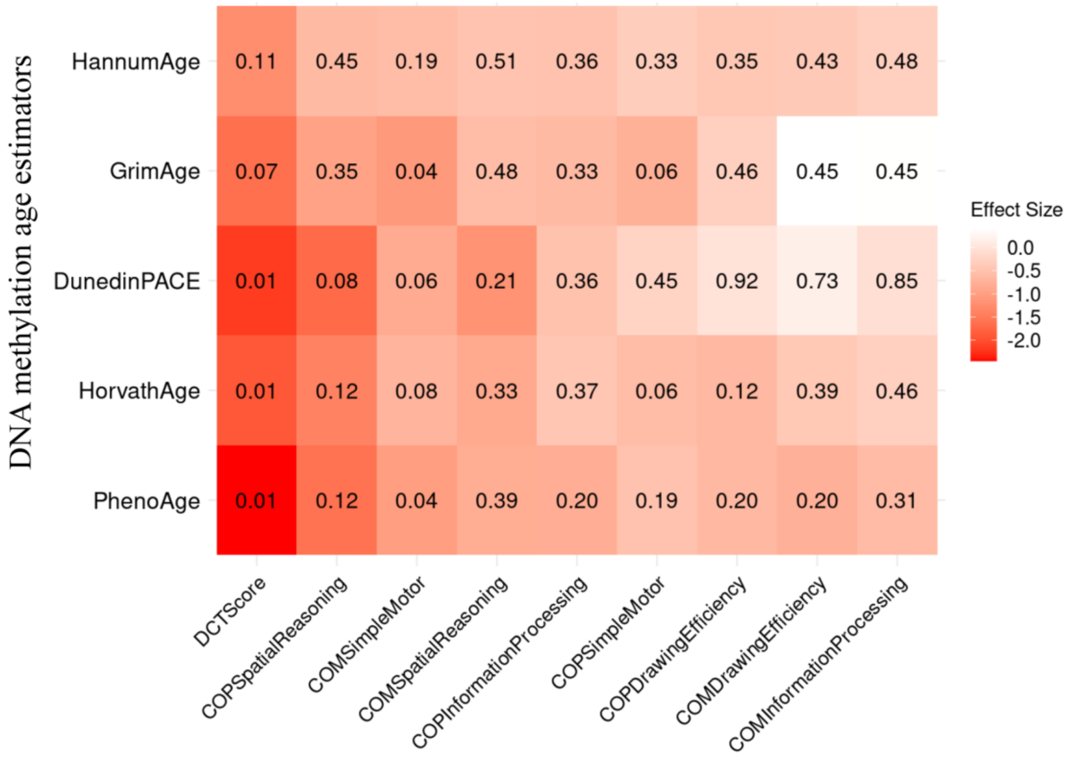

Supplementary Figure 2. Association between dCDT scores and DNAm age acceleration in elder age group (age ≥65). The dCDT total score includes command task composite scores and copy task composite scores. DNA methylation age acceleration was obtained by regressing DNAm age metrics on chronological age. We conducted association analysis between standardized DNAm age acceleration and the dCDT total score, adjusted for age, self−reported sex, education and cell counts. The numbers inside each cell represent the *P*−values of the associations. The color represents the change in dCDT scores corresponding to a one SD increase in DNAm age acceleration.

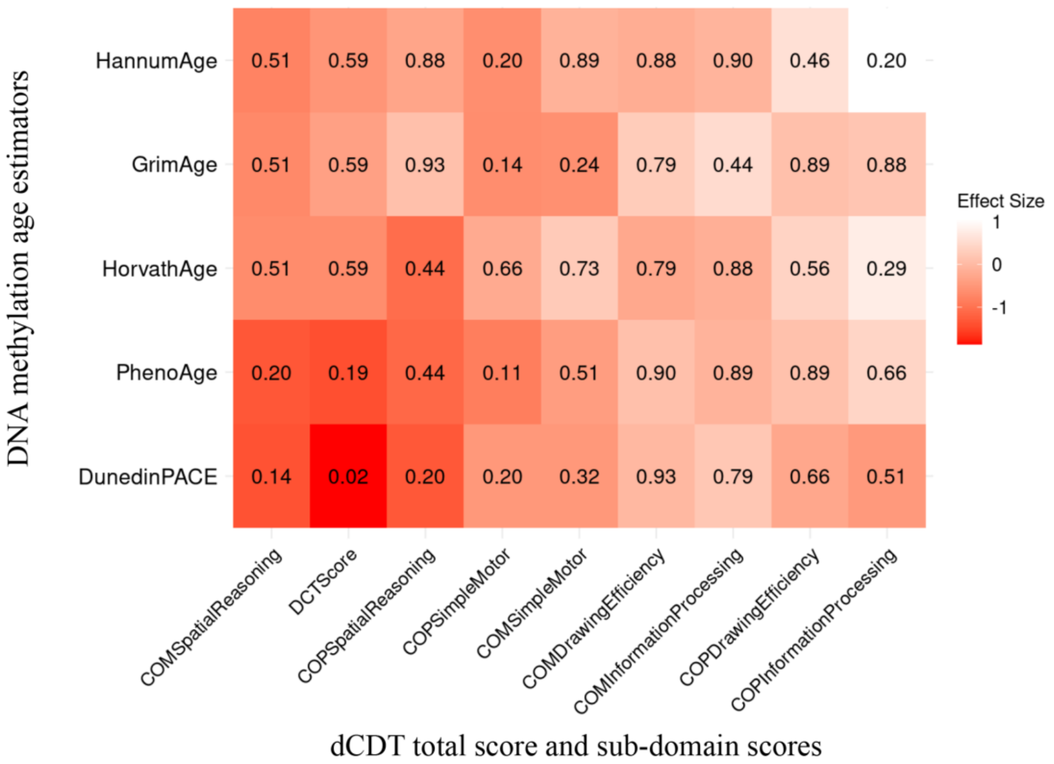

Supplementary Figure 3. Association between dCDT scores and DNAm age acceleration in younger age group (age <65). The dCDT total score includes command task composite scores and copy task composite scores. DNA methylation age acceleration was obtained by regressing DNAm age metrics on chronological age. We conducted association analysis between standardized DNAm age acceleration and the dCDT total score, adjusted for age, self−reported sex, education and cell counts. The numbers inside each cell represent the *P*−values of the associations. The color represents the change in dCDT scores corresponding to a one SD increase in DNAm age acceleration.

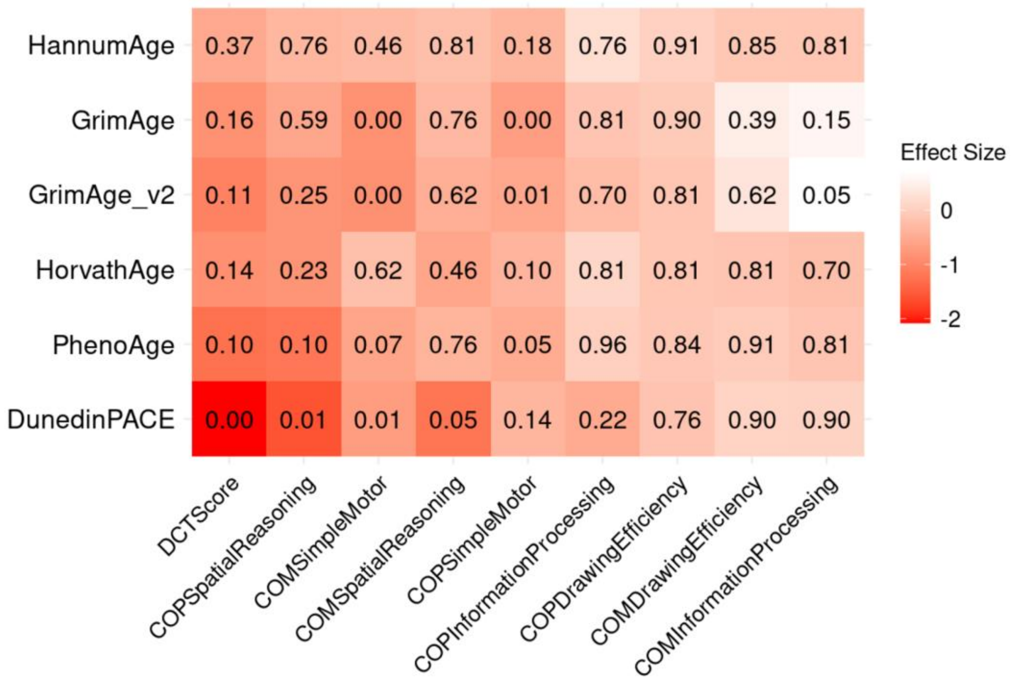

Supplementary Figure 4. Association between dCDT scores and DNAm age acceleration in the overall sample with GrimAge version two. The dCDT total score includes command task composite scores and copy task composite scores. DNA methylation age acceleration was obtained by regressing DNAm age metrics on chronological age. We conducted association analysis between standardized DNAm age acceleration and the dCDT total score, adjusted for age, self−reported sex, education and cell counts. The numbers inside each cell represent the *P*−values of the associations. The color represents the change in dCDT scores corresponding to a one SD increase in DNAm age acceleration.

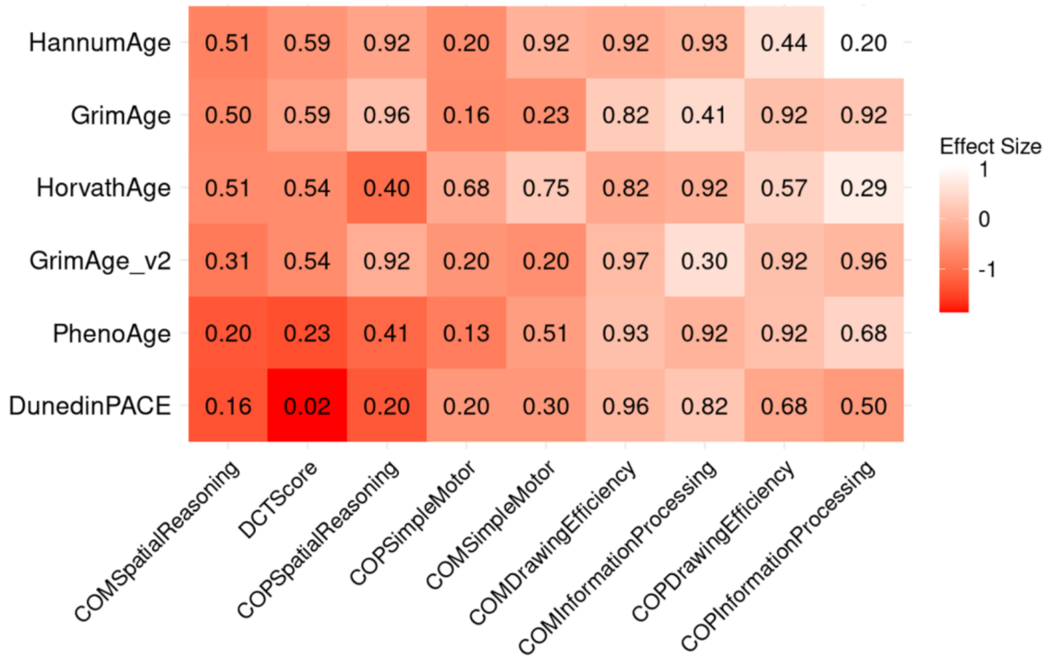

Supplementary Figure 5. Association between dCDT scores and DNAm age acceleration in the younger age group (age <65) with GrimAge version two. The dCDT total score includes command task composite scores and copy task composite scores. DNA methylation age acceleration was obtained by regressing DNAm age metrics on chronological age. We conducted association analysis between standardized DNAm age acceleration and the dCDT total score, adjusted for age, self−reported sex, education and cell counts. The numbers inside each cell represent the *P*−values of the associations. The color represents the change in dCDT scores corresponding to a one SD increase in DNAm age acceleration.

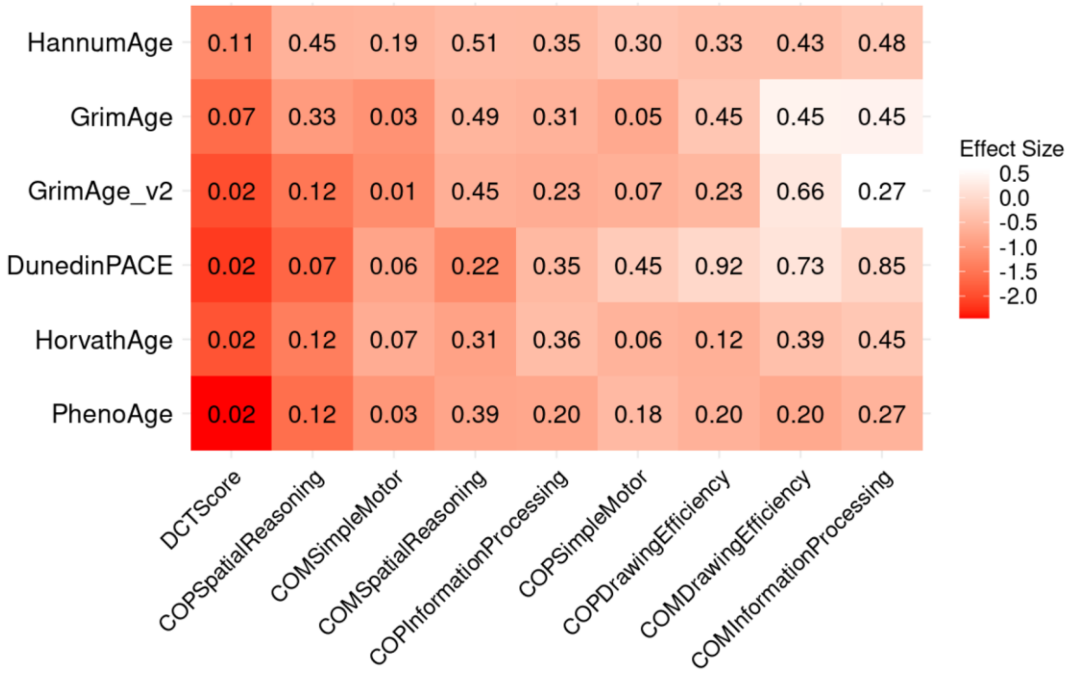

Supplementary Figure 6. Association between dCDT scores and DNAm age acceleration in the elder age group (age ≥65) with GrimAge version two. The dCDT total score includes command task composite scores and copy task composite scores. DNA methylation age acceleration was obtained by regressing DNAm age metrics on chronological age. We conducted association analysis between standardized DNAm age acceleration and the dCDT total score, adjusted for age, self−reported sex, education and cell counts. The numbers inside each cell represent the *P*−values of the associations. The color represents the change in dCDT scores corresponding to a one SD increase in DNAm age acceleration. The simple motor function from Copy task was significantly associated with GrimAge (FDR = 0.047).

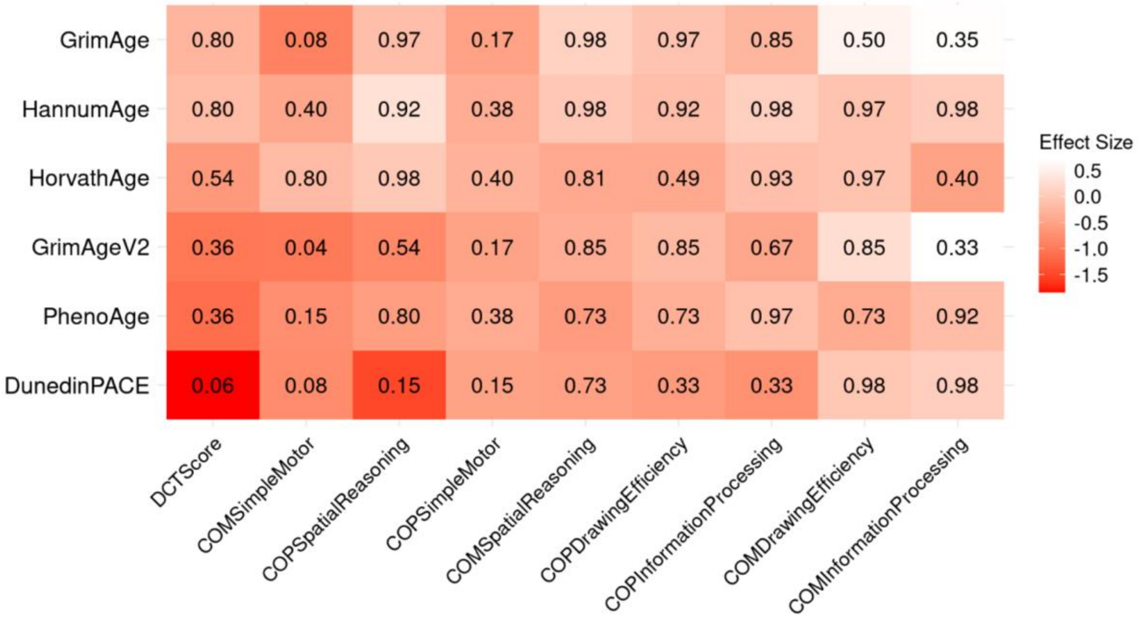

Supplementary Figure 7. Association between dCDT scores and DNAm age acceleration in the females. The dCDT total score includes command task composite scores and copy task composite scores. DNA methylation age acceleration was obtained by regressing DNAm age metrics on chronological age. We conducted association analysis between standardized DNAm age acceleration and the dCDT total score, adjusted for age, education and cell counts. The numbers inside each cell represent the *P*−values of the associations. The color represents the change in dCDT scores corresponding to a one SD increase in DNAm age acceleration.

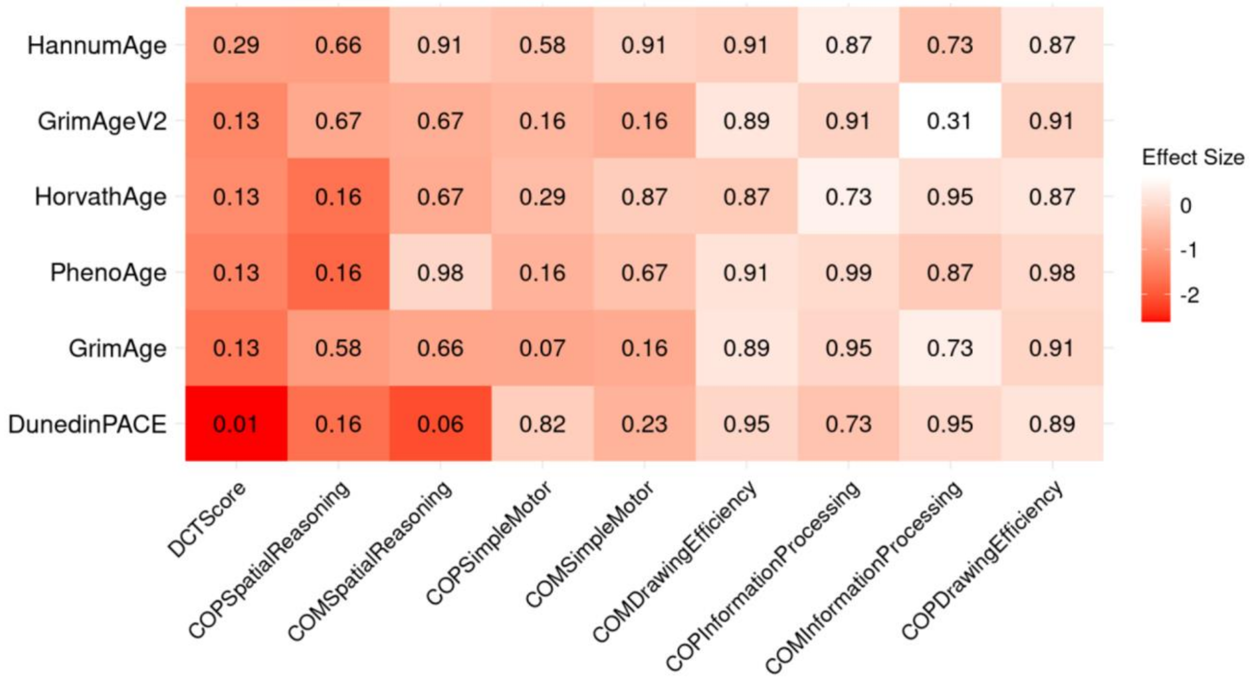

Supplementary Figure 8. Association between dCDT scores and DNAm age acceleration in the male participants. The dCDT total score includes command task composite scores and copy task composite scores. DNA methylation age acceleration was obtained by regressing DNAm age metrics on chronological age. We conducted association analysis between standardized DNAm age acceleration and the dCDT total score, adjusted for age, education and cell counts. The numbers inside each cell represent the *P*−values of the associations. The color represents the change in dCDT scores corresponding to a one SD increase in DNAm age acceleration.

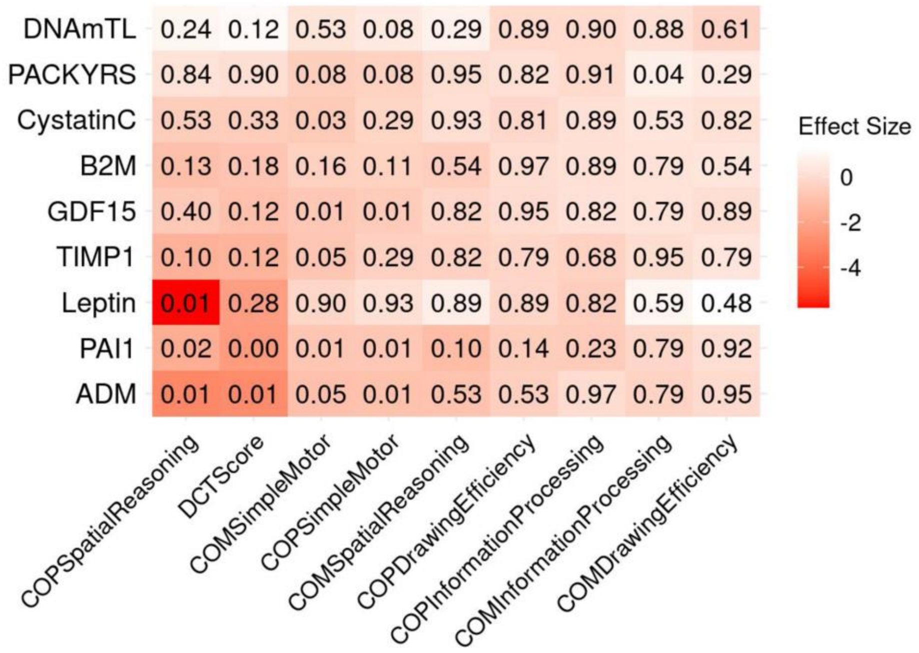

Supplementary Figure 9. Association between dCDT scores and DNA methylation−based plasma protein levels in the overall sample. The dCDT total score includes command task composite scores and copy task composite scores. Details about DNA methylation−based plasma protein levels can be found in a previous study [1]. We conducted association analysis between standardized DNAm plasma protein levels and the dCDT total score, adjusted for age, self−reported sex, and education. The numbers inside each cell represent the *P*−values of the associations. The color represents the change in dCDT scores corresponding to a one SD increase in DNAm plasma protein levels. ADM was significantly associated with simple motor function in the command task (beta = −1.0, FDR = 0.046).

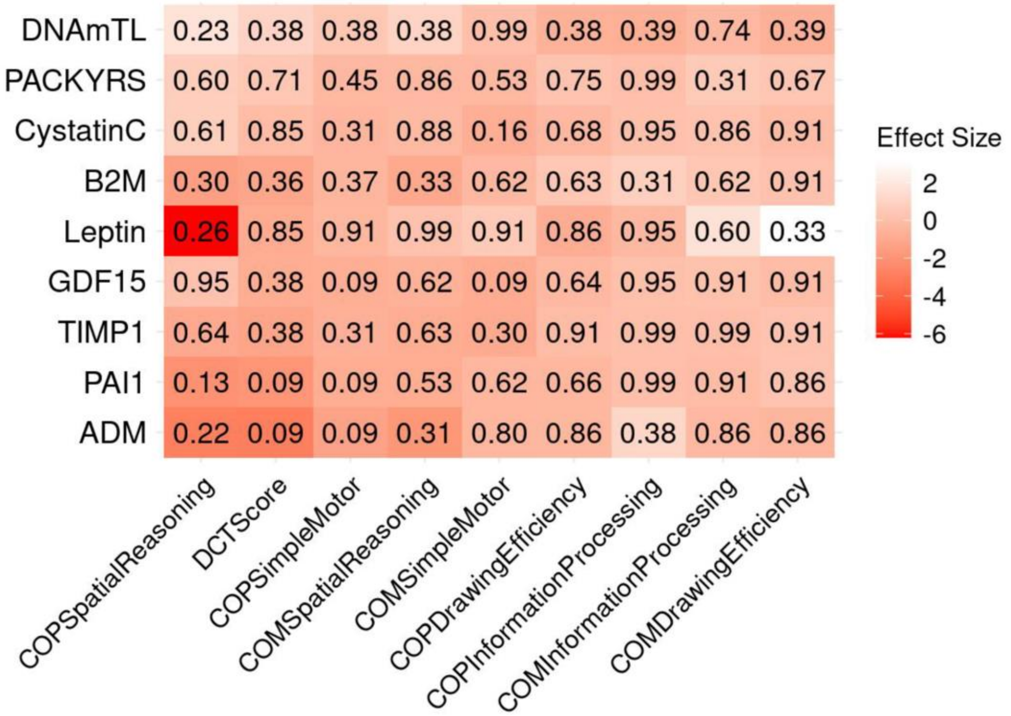

Supplementary Figure 10. Association between dCDT scores and DNA methylation−based plasma protein levels in the younger age group (age <65). The dCDT total score includes command task composite scores and copy task composite scores. Details about DNA methylation−based plasma protein levels can be found in a previous study [1]. We conducted association analysis between standardized DNAm plasma protein levels and the dCDT total score, adjusted for age, self−reported sex, and education. The numbers inside each cell represent the *P*−values of the associations. The color represents the change in dCDT scores corresponding to a one SD increase in DNAm plasma protein levels.

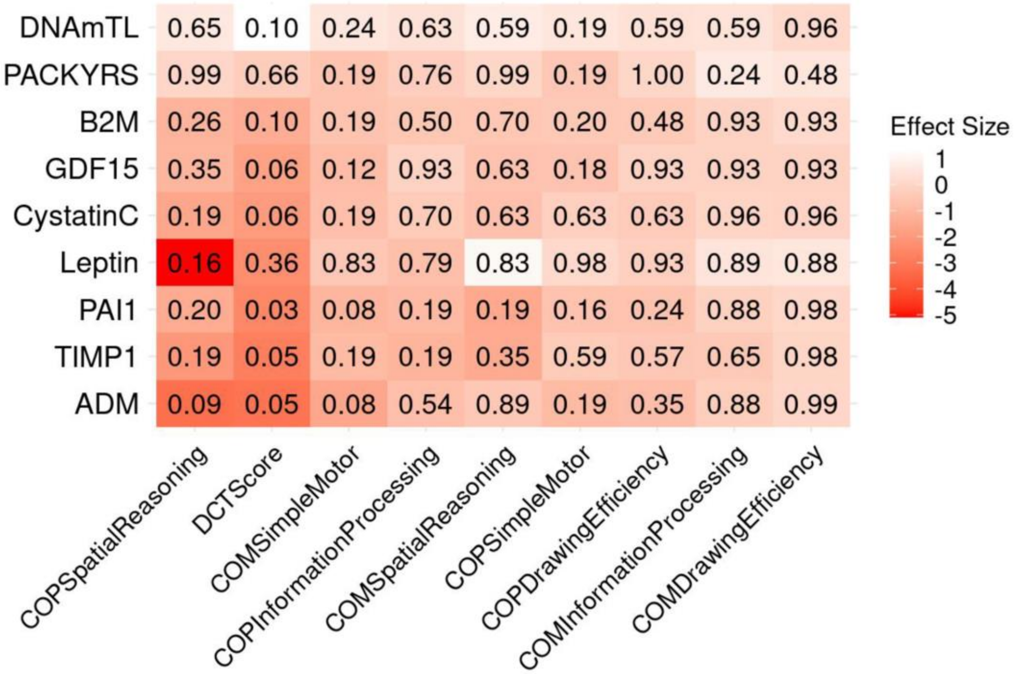

Supplementary Figure 11. Association between dCDT scores and DNAm −based plasma protein levels in the elder age group (age ≥65). The dCDT total score includes command task composite scores and copy task composite scores. Details about DNA methylation−based plasma protein levels can be found in a previous study [1]. We conducted association analysis between standardized DNAm plasma protein levels and the dCDT total score, adjusted for age, self−reported sex, and education. The numbers inside each cell represent the *P*−values of the associations. The color represents the change in dCDT scores corresponding to a one SD increase in DNAm plasma protein levels. dCDT total score significantly associated with PAI1 (beta = −2.1, FDR = 0.033), TIMP1 (beta = −2.8, FDR = 0.047), and ADM (beta = −2.8, FDR = 0.047).
