# Supplementary material for: Association of DNA methylation age acceleration with digital clock drawing test performance: the Framingham Heart Study
Source: Aging (Albany NY). 2025 Jul 21;17(7):1721–45. doi: 10.18632/aging.206285 (PMC12339028; doi:10.18632/aging.206285)
Supplement: Supplementary Tables [file aging-17-206285-s002.pdf]

SUPPLEMENTARY TABLES

Supplementary Table 1. Descriptive data of the three generations DNA methylation.

	Total	Age <65	Age ≥65
	**(*n* = 1789)**	**(*n* = 804)**	**(*n* = 985)**
Chronological age(years)	58 (± 12)	48 (± 7.3)	67 (± 6.5)
**First generation**			
Hannum	62 (± 9.0)	55 (± 6.3)	68 (± 5.8)
Horvath2	53 (± 7.9)	47 (± 5.6)	58 (± 5.7)
**Second generation**			
PhenoAge	52 (± 9.7)	45 (± 7.1)	59 (± 6.7)
GrimAge	69 (± 9.5)	61 (± 6.6)	75 (± 6.0)
**Third generation**			
DunedinPACE	1.1 (± 0.12)	1.0 (± 0.11)	1.1 (± 0.11)

The chronological age, first−generation and second−generation epigenetic clock measure age in years. The third−generation epigenetic clock measure aging in pace of aging. Mean with standard deviation is reported for each cell.

Supplementary Table 2. Descriptive data of advance aging in different sex groups.

	Total	Male	Female
	**(*n* = 1789)**	**(*n* = 834)**	**(*n* = 955)**
**First Generation**			
Hannum	49.6	66.2	35.1
Horvath	49.1	56.2	42.8
**Second Generation**			
PhenoAge	47.6	53.7	42.3
GrimAge	40.4	57.4	25.4
**Third Generation**			
DunedinPACE	65.0	67.6	62.7

This table shows the percentage of participants with advanced aging.

Supplementary Table 3. Interaction analysis between epigenetic age residual and age at dDCT age.

	Coefficient of interaction term	Coefficient of DNAm age residual	Coefficient of age at dCDT	*P*−Value of interaction term
DunedinPACE	−0.9	41.6	0.42	0.004*
Hannum	−0.002	−0.06	−0.50	0.880
Horvath	−0.009	0.40	−0.50	0.302
PhenoAge	−0.0007	−0.23	−0.50	0.931
GrimAge	0.001	−0.40	−0.50	0.928

Supplementary Table 4. Interaction analysis between epigenetic age residual and sex.

	Coefficient of interaction term	Coefficient of DNAm age residual	Coefficient of sex	*P*−Value of interaction term
DunedinPACE	−0.3	−17.0	4.00	0.967
Hannum	−0.004	−0.16	4.04	0.989
Horvath	0.04	−0.30	4.20	0.844

PhenoAge	−0.04	−0.20	4.04	0.835
GrimAge	0.13	−0.50	3.65	0.629

Supplementary Table 5. Association analysis between dCDT scores and DNA methylation−based plasma protein levels in GrimAge estimation.

	Total	Age < 65	Age ≥65
	**(*n* = 1789)**	**(*n* = 804)**	**(*n* = 985)**
**dCDTScore**			
ADM	−2.86 (−4.69, −1.04)	−2.93 (−5.38, −0.49)	−3.11 (−5.63, −0.59)
B2M	−0.87 (−1.95, 0.21)	−1.13 (−2.55, 0.28)	−1.37 (−2.88, 0.14)
CystatinC	−0.58 (−1.67, 0.50)	−0.12 (−1.41, 1.16)	−1.94 (−3.69, −0.20)
GDF15	−1.02 (−2.10, 0.06)	−0.86 (−2.24, 0.52)	−1.73 (−3.33, −0.13)
Leptin	−2.25 (−5.80, 1.29)	−0.88 (−6.63, 4.84)	−2.33 (−6.86, 2.20)
PAI1	−2.33 (−3.53, −1.13)	−1.91 (−3.49, −0.34)	−2.54 (−4.20, −0.88)
TIMP1	−1.47 (−3.00, 0.05)	−1.22 (−3.29, 0.85)	−2.82 (−4.96, −0.68)
**COPDrawingEfficiency**			
ADM	−0.49 (−1.30, 0.31)	−0.36 (−1.60, −0.87)	−0.81(−1.85, 0.23)
B2M	0.01 (−0.47, 0.49)	0.36 (−0.35, 1.08)	−0.42 (−1.04, 0.21)
CystatinC	−0.16 (−0.64, 0.32)	−0.28 (−0.94, 0.37)	−0.35 (−1.07, 0.37)
GDF15	−0.03 (−0.51, 0.44)	−0.34 (−1.04, 0.36)	−0.11 (−0.78, 0.55)
Leptin	−0.26 (−1.83, 1.30)	−0.81 (−3.69, 2.05)	−0.25 (−2.13, 1.63)
PAI1	−0.56 (−1.09, −0.03)	−0.37 (−1.17, 0.43)	−0.64 (−1.32, 0.05)
TIMP1	−0.25 (−0.92, 0.42)	−0.17 (−1.21, 0.87)	−0.51 (−1.40, 0.38)
**COPSimpleMotor**			
ADM	−0.99 (−1.57, −0.40)	−1.30 (−2.18, −0.43)	−0.94 (−1.73, −0.15)
B2M	−0.39 (−0.74, −0.04)	−0.43 (−0.94, 0.07)	−0.46 (−0.94, 0.01)
CystatinC	−0.29 (−0.64, 0.06)	−0.46 (−0.93, 0.01)	−0.25 (−0.80, 0.29)
GDF15	−0.60 (−0.95, −0.25)	−0.78 (−1.28, −0.28)	−0.61 (−1.12, −0.11)
Leptin	0.12 (−1.03, 1.26)	−0.39 (−2.43, 1.65)	0.08 (−1.34, 1.50)
PAI1	−0.74 (−1.13, −0.35)	−0.82 (−1.38, −0.25)	−0.66 (−1.18, −0.14)
TIMP1	−0.42 (−0.91, 0.07)	−0.70 (−1.45, 0.04)	−0.37 (−1.04, 0.31)
**COPInformationProcessing**			
ADM	−0.02 (−1.00, 0.97)	1.16 (−0.29, −2.61)	−0.80 (−2.12, 0.52)
B2M	0.11 (−0.49, 0.69)	0.80 (−0.04, 1.64)	−0.50 (−1.30, 0.29)
CystatinC	0.12 (−0.47, 0.69)	0.07 (−0.70, 0.85)	−0.37 (−1.28, 0.55)
GDF15	0.17 (−0.41, 0.76)	0.09 (−0.73, 0.91)	−0.11 (−0.95, 0.73)
Leptin	−0.53 (−2.45, 1.38)	−0.28 (−3.66, 3.10)	−0.77 (−3.14, 1.60)
PAI1	−0.60 (−1.25, 0.05)	−0.004 (−0.93, 0.94)	−0.94 (−1.81, −0.07)
TIMP1	−0.38 (−1.20, 0.44)	0.04 (−1.19, 1.27)	−1.15 (−2.27, −0.03)
**COPSpatialReasoning**			
ADM	−3.02 (−4.65, −1.39)	−2.86 (−5.29, −0.44)	−3.23 (−5.41, −1.06)
B2M	−1.04 (−2.01, −0.08)	−1.44 (−2.85, −0.04)	−1.14 (−2.45, 0.17)
CystatinC	−0.58 (−1.55, 0.39)	−0.74 (−0.54, 2.03)	−1.59 (−3.10, −0.07)
GDF15	−0.70 (−1.68, 0.27)	0.13 (−1.24, 1.51)	−1.07 (−2.47, 0.32)
Leptin	−5.79 (−8.95, −2.63)	−6.21 (−11.87, −0.58)	−5.09 (−9.01, −1.17)
PAI1	−1.71 (−2.78, −0.63)	−2.10 (−3.67, −0.54)	−1.42 (−2.86, 0.02)
TIMP1	−1.57 (−2.93, −0.21)	−0.99 (−3.05, 1.06)	−2.05 (−3.91, −0.19)

**COMDrawingEfficiency**			
ADM	−0.05 (−1.04, 0.93)	−0.42 (−1.91, 1.07)	−0.04 (−1.33, 1.25)
B2M	0.33 (−0.25, 0.91)	0.15 (−0.72, 1.01)	0.12 (−0.65, 0.89)
CystatinC	0.16 (−0.42, 0.75)	−0.11 (−0.90, 0.67)	−0.08 (−0.97, 0.82)
GDF15	0.10 (−0.49, 0.68)	−0.13 (−0.97, 0.72)	−0.11 (−0.93, 0.71)
Leptin	1.27 (−0.65, 3.18)	3.09 (−0.39, 6.56)	0.54 (−1.77, 2.85)
PAI1	0.08 (−0.57, 0.72)	0.25 (−0.71, 1.22)	0.04 (−0.81, 0.89)
TIMP1	0.29 (−0.54, 1.11)	0.19 (−1.07, 1.45)	−0.07 (−1.17, 1.02)
**COMInformationProcessing**			
ADM	−1.00 (−1.74, −0.25)	−0.38 (−1.48, 0.71)	−1.58 (−2.58, −0.58)
B2M	−0.45 (−0.89, −0.01)	−0.35 (−0.98, 0.28)	−0.64 (−1.24, −0.04)
CystatinC	−0.65 (−1.09, −0.21)	−0.75 (−1.33, −0.16)	−0.79 (−1.49, −0.10)
GDF15	−0.78 (−1.22, −0.34)	−0.89 (−1.51, −0.27)	−0.89 (−1.53, −0.25)
Leptin	−0.22 (−1.67, 1.22)	0.50 (−2.04, 3.04)	−0.52 (−2.32, 1.29)
PAI1	−0.83 (−1.32, −0.34)	−0.38 (−1.09, 0.33)	−1.07 (−1.74, −0.41)
TIMP1	−0.83 (−1.45, −0.21)	−0.95 (−1.88, −0.02)	−0.94 (−1.79, −0.08)
**COMSpatialReasoning**			
ADM	−0.33 (−1.20, 0.54)	−0.31 (−1.52, 0.90)	−0.30 (−1.51, 0.91)
B2M	0.19 (−0.32, 0.71)	0.37 (−0.33, 1.08)	−0.12 (−0.85, 0.60)
CystatinC	0.32 (−0.20, 0.84)	0.17 (−0.47, 0.82)	−0.08 (−0.91, 0.76)
GDF15	0.20 (−0.32, 0.72)	0.13 (−0.56, 0.81)	−0.13 (−0.90, 0.64)
Leptin	0.89 (−0.80, 2.57)	1.67 (−1.15, 4.48)	0.46 (−1.72, 2.63)
PAI1	−0.20 (−0.77, 0.37)	−0.13 (−0.91, 0.65)	−0.19 (−0.99, 0.61)
TIMP1	0.03 (−0.69, 0.76)	−0.01 (−1.03, 1.01)	−0.45 (−1.47, 0.58)
**COPSpatialReasoning**			
ADM	−0.94 (−2.48, 0.60)	−1.86 (−3.85, 0.13)	−0.48 (−2.70, 1.72)
B2M	−0.52 (−1.44, 0.39)	−1.04 (−2.19, 0.11)	−0.52 (−1.84, 0.80)
CystatinC	0.08 (−0.83, 1.00)	−0.25 (−1.30, 0.81)	−0.72 (−2.25, 0.81)
GDF15	−0.25 (−1.17, 0.66)	−0.61 (−1.73, 0.52)	−0.67 (−2.07, 0.74)
Leptin	0.62 (−2.37, 3.61)	0.13 (−4.53, 4.77)	1.14 (−2.82, 5.10)
PAI1	−1.18 (−2.20, −0.17)	−0.83 (−2.12, 0.45)	−1.46 (−2.92, −0.01)
TIMP1	−0.38 (−1.67, 0.91)	−0.87 (−2.55, 0.81)	−1.44 (−3.31, 0.44)

Note: Effect size with 95% confidence intervals is provided for each plasma protein level. Subdomain scores of dCDT copy task start with COP; subdomain scores dCDT command task start with COM. Detailed information about how each plasma protein level was used in GrimAge estimation can be found in a previous study [1].

SUPPLEMENTARY REFERENCE

1. Lu AT, Quach A, Wilson JG, Reiner AP, Aviv A, Raj K, Hou L, Baccarelli AA, Li Y, Stewart JD, Whitsel EA, Assimes TL, Ferrucci L, Horvath S. DNA methylation GrimAge strongly predicts lifespan and healthspan. Aging (Albany NY). 2019; 11:303–27. https://doi.org/10.18632/aging.101684 PMID:30669119
